# Pressurized intraperitoneal aerosolized chemotherapy (PIPAC) experience in patients with recurrent low grade serous ovarian carcinoma (LGSOC): sub-cohort report of phase 1 clinical trial

**DOI:** 10.3389/fonc.2024.1404936

**Published:** 2024-08-01

**Authors:** Brad Nakamura, Rosemary Senguttuvan, Nora H. Ruel, Paul H. Frankel, Susan E. Yost, Sarah Cole, Sue Chang, Alexander Jung, Melissa Eng, Raechelle Tinsley, Daphne Stewart, Edward Wang, Joshua Cohen, Jeannine Villella, Richard L. Whelan, Amit Merchea, Danielle K. DePeralta, Mihaela Cristea, Mark T. Wakabayashi, Mustafa Raoof, Thanh Hue Dellinger

**Affiliations:** ^1^ Department of Surgery, City of Hope National Medical Center, Duarte, CA, United States; ^2^ Department of Computation and Quantitative Medicine, City of Hope National Medical Center, Duarte, CA, United States; ^3^ Department of Medical Oncology and Therapeutics Research, City of Hope National Medical Center, Duarte, CA, United States; ^4^ Clinical Protocol Development, City of Hope National Medical Center, Duarte, CA, United States; ^5^ Department of Pathology, City of Hope National Medical Center, Duarte, CA, United States; ^6^ Department of Diagnostic Radiology, City of Hope National Medical Center, Duarte, CA, United States; ^7^ Department of Surgery, Northwell Health, New York, NY, United States; ^8^ Department of Surgery, Mayo Clinic Florida, Jacksonville, FL, United States; ^9^ Regeneron Pharmaceuticals, Tarrytown, NY, United States

**Keywords:** low-grade serous ovarian carcinoma, LGSOC, pressurized intraperitoneal aerosolized chemotherapy, PIPAC, recurrent

## Abstract

**Introduction:**

Low grade serous ovarian carcinoma (LGSOC) is a rare subtype of ovarian cancer (OC) that is challenging to treat due to its relative chemoresistance. Given that LGSOC patients often recur in the peritoneal cavity, novel intraperitoneal (IP) chemotherapy should be explored. Pressurized intraperitoneal aerosolized chemotherapy (PIPAC) is a method that has demonstrated peritoneal disease control in cancers with peritoneal metastases.

**Methods:**

NCT04329494 is a US multicenter phase 1 trial evaluating the safety of PIPAC in recurrent ovarian, uterine, and GI cancers with peritoneal metastases. This analysis describes the outcomes of a sub-cohort of four LGSOC patients treated with IP cisplatin 10.5 mg/m^2^, doxorubicin 2.1 mg/m^2^ PIPAC q4-6 weeks. Primary endpoints included dose-limiting toxicities (DLT) and incidence of adverse events (AE). Secondary endpoints were progression free survival (PFS) and treatment response based on radiographic, intraoperative, and pathological findings.

**Results:**

Four patients with LGSOC were enrolled of which three were heavily pretreated. Median prior lines of therapy was 5 (range 2-10). Three patients had extraperitoneal metastases, and two patients had baseline partial small bowel obstructive (SBO) symptoms. Median age of patients was 58 (38-68). PIPAC completion rate (≥2 PIPACs) was 75%. No DLTs or Clavien-Dindo surgical complications occurred. No G4/G5 AEs were observed, and one G3 abdominal pain was reported. One patient had a partial response after 3 cycles of PIPAC and completed an additional 3 cycles with compassionate use amendment. Two patients came off study after 2 cycles due to extraperitoneal progressive disease. One patient came off study after 1 cycle due to toxicity. Median decrease in peritoneal carcinomatosis index between cycles 1 and 2 was 5.0%. Ascites decreased in 2 out of 3 patients who had ≥2 PIPACs. Median PFS was 4.3 months (1.7-21.6), median overall survival was 11.6 months (5.4-30.1), and objective response rate was 25%.

**Conclusion:**

PIPAC with cisplatin/doxorubicin is well tolerated in LGSOC patients without baseline SBO symptoms. IP response was seen in 2 out of 3 patients that completed ≥2 PIPAC cycles. Further study of PIPAC for patients with recurrent disease limited to the IP cavity and with no partial SBO symptoms should be considered.

## Introduction

Low-grade serous ovarian carcinoma (LGSOC) is a rare subtype of epithelial ovarian cancer (OC). It accounts for 2-5% of all epithelial OC and 4.7% of all serous OC (1). LGSOC is rarely associated with BRCA mutations or family histories of breast or OC (2). Compared to women diagnosed with common high-grade serous ovarian carcinoma (HGSOC), women with LGSOC often have a longer disease trajectory but experience fewer disease-free intervals. Thus, LGSOC patients often receive numerous treatment regimens in a continuous fashion, while women with HGSOC may experience several intervals of time in clinical remission allowing for time off treatment. Of women with advanced-stage LGSOC, 70% will experience a disease recurrence. When possible, obtaining a commercially available somatic mutation profile may be considered to identify the best treatment targets. Multiple options exist in this setting including secondary cytoreductive surgery, chemotherapy, endocrine/hormonal therapies, targeted agents, and clinical trials (3).

The peritoneum is one of the primary sites of metastasis and recurrence, often resulting in malignant gastro-intestinal and urinary obstruction, and reduced quality of life (QoL), and significant morbidity in LGSOC patients. These peritoneal metastases are frequently unresectable and refractory to systemic therapy due to pharmacokinetic limitations, poor peritoneal drug uptake, and impaired local drug distribution (4).

Treatment options targeting the peritoneum have not been extensively studied in this population, and innovative combinations that consider tumor biology and peritoneal metastases are urgently needed. Regional therapy offers a pharmacokinetic advantage with improved peritoneal to plasma drug ratios and has proven to be effective in epithelial OC (5). IP chemotherapy has demonstrated survival advantages for OC patients with both normothermic IP chemotherapy and hyperthermic IP chemotherapy (HIPEC). As LGSOC is a rare OC subtype, limited patients with this disease were enrolled in the GOG 172 IP chemotherapy trial (5) or in the OVHIPEC-1 trial (2). Nonetheless, both IP chemotherapy and HIPEC treatments are limited to newly diagnosed OC patients during first-line therapy, and not in the recurrent setting. The role of IP chemotherapy in recurrent epithelial OC has been limited due to the need for optimal cytoreduction for both IP chemotherapy and HIPEC.

**Figure 1:**
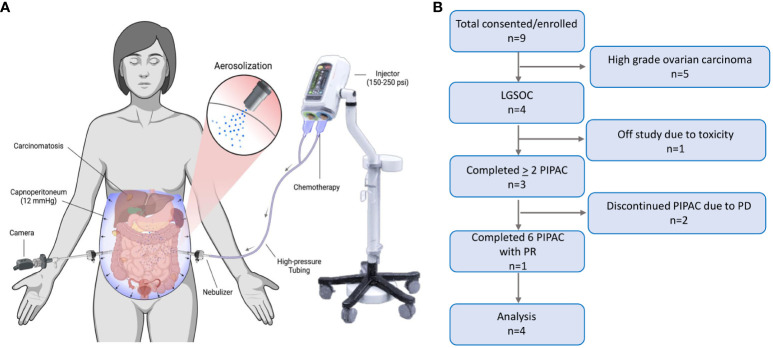
Pressurized Intraperitoneal Aerosolized Chemotherapy. **(A)** PIPAC is a laparoscopic chemotherapy delivery method for superior drug delivery to peritoneal metastases. It improves drug distribution through aerosolization of chemotherapy in the abdominal cavity, via a nebulizer. It improves drug tissue absorption through pressurization of the drug via a 12 mmHg capnoperitoneum induced by a high-pressure injector (BioRender.com). **(B)** Consolidated Standards of Reporting Trials (CONSORT) flow diagram of the progression of patients through the trial, including consent, enrollment, treatment completed, follow-up, and analysis. LGSOC, low grade serous ovarian carcinoma; PIPAC, pressurized intraperitoneal aerosolized chemotherapy; PD, progressive disease; PR, partial response.

Pressurized intraperitoneal aerosolized chemotherapy (PIPAC) is a novel treatment modality that intensifies chemotherapy delivery to peritoneal metastases to improve drug distribution and penetration of peritoneal tumors (6). It does so via aerosolization of chemotherapy into gas-like microdroplets through a micropump delivered via a high-pressure injector. This chemotherapy administration occurs during the creation of temporary intra-abdominal pressure using CO_2_ gas administered during laparoscopic surgery (**Figure 1A**), at routine pressures of 12 mm Hg applied for a 30-minute duration. The increased intra-abdominal pressure helps to overcome the interstitial pressure within the tumor, which is one of the barriers exerted by the fluid within the tumor tissue that limits the penetration of conventional chemotherapy drugs.

In comparison to HIPEC, PIPAC does not require cytoreduction, can be frequently repeated, and is well tolerated. The clinical efficacy and safety of PIPAC in OC has been studied in multiple, international phase I and phase II trials over the past decade. The need for standardization of PIPAC protocols has been highlighted with the development of recommendations based on expert panel consensus and in person courses established by the International Society for the Study of Pleura and Peritoneum (7–9). Based on this expert panel consensus meeting in 2021, an optimal dose for the combination of cisplatin 10.5 mg/m^2^ and doxorubicin 2.1 mg/m^2^ was established based on safety and efficacy data from prior clinical trials including 2 phase I dose-escalation studies showing no difference in local or systemic toxicities between varying doses of cisplatin (7.5-30 mg/m^2^) and doxorubicin (1.5-6 mg/m^2^) (10, 11). In both phase I dose-escalation trials, the maximum tolerated doses were not reached. Of note, the Robella et al., 2021 study demonstrated a much higher tolerable dose, up to cisplatin 6 mg/m^2^ with doxorubicin 30 mg/m^2^, however this was administered as a single dose of PIPAC in this trial (11). Two recent retrospective studies, the systemic review by Taliento et al., 2023 and the multicenter cohort study by Kefleyesus et al., 2023 demonstrated the safety and encouraging efficacy results in a select population of ovarian cancer patients using the combination of PIPAC cisplatin 7.5 mg/m^2^ with doxorubicin 1.5 mg/m^2^ and cisplatin 10.5 mg/m^2^ with doxorubicin 2.1 mg/m^2^ (12, 13). Thus, more studies are needed to establish the optimal dose of this combination of drugs used in PIPAC.

Currently, there is an ongoing, open-label, randomized phase III trial, CTRI2018/08/021223 in India, comparing PIPAC versus IV chemotherapy in platinum-resistant recurrent OC patients (14). Preliminary data of this trial comparing 3 cycles of PIPAC cisplatin 15 mg/m^2^ and doxorubicin 3 mg/m^2^ versus 6 cycles of single agent IV chemotherapy has shown an objective response rate (ORR) of 66.6% versus 22.5% respectively with fewer grade 3-4 adverse events, 10.0% versus 35.7% respectively (15).

This study is the first PIPAC clinical trial in the U.S. and is being conducted as an open label U.S. multicenter phase I trial (NCT04329494). As LGSOC is a rare OC subtype, limited data exists on PIPAC in this population, and clinical trials have focused on OC of all subtypes. Here, we present preliminary data of a sub-cohort of LGSOC patients from arm 1 of this ongoing clinical trial.

## Materials and methods

### Ethics statement

This study was conducted according to the principles of the Belmont Report: Ethical Principles and Guidelines for the Protection of Human Subjects or Research and the Declaration of Helsinki. All patients completed written documentation of informed consent to participate. This consent included the use of data and images for publication. This study was approved by the City of Hope Institutional Review Board (IRB) (#19184), the Northwell Health IRB (#20-0859), and the Mayo Clinic IRB (#20-010121).

### Patients

Adult patients ≥ 18 years old with histologically confirmed invasive LGSOC with peritoneal carcinomatosis who had progressed on at least one prior standard chemotherapeutic regimen were included if they had Eastern Cooperative Oncology Group (ECOG) performance status ≤ 2, no contraindications to laparoscopic surgery or aerosol therapy, intraoperative laparoscopic findings showing PIPAC access is feasible, no evidence of impending bowel obstruction, ≤ 5L of ascites, and patient is not a candidate for cytoreduction and HIPEC. Exclusion criteria included prior treatment with maximum cumulative doses of anthracyclines and/or anthracenediones. See **Supplementary Table 1** for complete eligibility criteria.

### Study design

This is an ongoing, phase I clinical trial without dose escalation to establish the safety of cisplatin 10.5 mg/m^2^ PIPAC and doxorubicin 2.1 mg/m^2^ PIPAC. The rules for accrual were slot-limited to not exceed the risk of the traditional 3 + 3 phase I trial design with modifications to adapt to the patient queue to reduce the time to complete the study (16, 17). If the proposed treatment had not been well-tolerated, the plan was to amend the study. Prior to instillation of PIPAC during each procedure, ascites was suctioned and measured, visual assessment of tumor burden was recorded via Peritoneal Carcinomatosis Index (PCI), and biopsies were obtained from all 4 quadrants if accessible to assess peritoneal regression grading score (PRGS) (18). Selection of biopsy sites in each quadrant was based on surgeon evaluation of largest and most suspicious appearing tumor lesion. The PIPAC procedure was performed with IP cisplatin 10.5 mg/m^2^ in 150 mL NaCl 0.9% and doxorubicin 2.1 mg/m^2^ in 50 mL NaCl 0.9% delivered using a high-pressure injection (Medrad Stellant injector, Bayer Corporation) and Capnopen nebulizer (Capnomed Corporation, Tubingen, Germany and REGER Medizintechnik GmbH, Villingendorf, Germany) at a maximum of 300 psi and 30mL/min, followed by a 30-min pneumoperitoneum at 12 mmHg containing the aerosolized chemotherapy at room temperature prior to release of the pneumoperitoneum. Laparoscopic balloon occlusion ports were used for staff safety. Standardized left lower quadrant port placement was used for PIPAC delivery unless it was not safely feasible. Limited adhesiolysis was allowed, however no other surgical interventions or resection of tumors were performed. PIPAC cisplatin and doxorubicin were given every 4-6 weeks for a total of three treatments provided that no severe AE, dose-limiting toxicity (DLT), disease progression, or patient withdrawal occurred. DLTs were defined as any delay greater than 21 days; any grade 3 or higher nonhematologic toxicity excluding grade 3 nausea, vomiting, abdominal pain, or diarrhea adequately treated that returns to grade 2 or less within 48 hours; grade 3 fatigue that returns to grade 2 or less within 7 days; grade 3 laboratory/metabolic abnormalities that are not considered clinically significant and are easily correctable to grade 2 or less within 72 hours; grade 3 infusion-related reaction (first occurrence and in the absence of steroid prophylaxis) that resolves within 6 hours with appropriate clinical management; and grade 3 peripheral neuropathy. Additional DLTs include Clavien-Dindo grade IIIB or higher surgical complications; grade 4 thrombocytopenia or neutropenia lasting more than 7 days or associated with fever or infection. Quality of life (QOL) measures were collected via patient surveys. Patients with clinical benefit were offered additional PIPAC cycles on compassionate care.

This paper describes the data analysis up to January 2024 of this ongoing clinical trial. The last LGSOC patient in this sub-cohort was enrolled in February 2023.

### Endpoints

The primary endpoints were DLTs and incidence of treatment related AEs. AEs were assessed every 4-6 weeks using Common Terminology Criteria for Adverse Events (CTCAE v5.0) for up to 18 weeks. Follow-up after treatment completion (≥2 PIPACs) was every 12 weeks. Secondary endpoints included PFS and treatment response. Treatment response was based on changes in computed tomography (CT) imaging Response Evaluation Criteria in Solid Tumors (RECIST) version 1.1, intraoperative PCI and pathologic PRGS of multiple biopsies taken each cycle.

### Statistical analysis

Simple mathematical ratios, medians, and ranges are reported. Measurements of association and statistical significance were not calculated given a limited sample size.

### Micropump device

The micropump used for chemotherapy delivery is a Class III, Category A nebulizer device, and an investigational new drug (IND) combination product application by City of Hope (COH). The U.S. Food and Drug Administration (FDA) approved the study (IND/IDE 147749) in 2020. In this study, high-pressure micro-injection pump (MIP) is interchangeable with nebulizer.

## Results

### Patient characteristics

Nine recurrent epithelial OC patients were enrolled, of which four had LGSOC (**Figure 1B**). The median age of LGSOC patients was 58 years (range 38-68) (**Table 1**). Three (75%) patients had good performance status with ECOG score 1, and one patient had ECOG score 2. LGSOC patients were heavily pretreated, with median prior lines of therapy of 5 (range 2-10). At baseline, three (75%) patients had extraperitoneal metastases, and two (50%) patients had baseline partial small bowel obstructive (SBO) symptoms. The median baseline PCI was 20 and the median PRGS was 2.75. The volume of ascites at time of first PIPAC cycle for each patient was 10 cc, 50 cc, 1500 cc, and 3000 cc. **Supplementary Table 2** displays de-identified individual patient data.

**Table 1:** LGSOC patient characteristics, response, and survival.

Characteristic	N=4
Age, years^1^	58 (38-68)
Race/Ethnicity	
Non-Hispanic White	4 (100%)
Hispanic White	0 (0%)
ECOG	
1	3 (75%)
2	1 (25%)
Prior lines of therapy^1^	5 (2-10)
Baseline metastatic sites	
IP only	1 (25%)
Extraperitoneal and IP	3 (75%)
Patients with ≥2 PIPAC cycles	3 (75%)
Baseline PCI^1^	20 (20-33)
Baseline PRGS^1^	2.75 (1.75-3.50)
Baseline ascites volume	
Large volume (≥500cc)	2 (50%)
Small volume (<500cc)	2 (50%)
Not present	0 (0%)
Best response per RECIST	
PR	1 (25%)
SD	1 (25%)
PD	1 (25%)
Unknown^2^	1 (25%)
Percent change in PCI from cycle 1 to 2 for patients receiving ≥2 cycles^1^	-5% (-30% - +15%)
PFS, months^1^	4.3 (1.7-21.6)
OS, months^1^	11.6 (5.4-30.1)
Off treatment reason	
Progression	2 (50%)
Toxicity	1 (25%)
Treatment complete	1 (25%)
Progression type	
IP only	1 (25%)
Extraperitoneal and IP	2 (50%)
Unknown	1 (25%)

^1^Median (range); ^2^No follow-up imaging after 1 cycle of PIPAC; ECOG, Eastern Cooperative Oncology Group; IP, intraperitoneal; PIPAC, pressurized intraperitoneal aerosolized chemotherapy; PCI, peritoneal carcinomatosis index; PRGS, peritoneal regression grading system; RECIST, Response Evaluation Criteria in Solid Tumors; PFS, progression free survival; OS, overall survival

### Feasibility of PIPAC

There were no technical failures in completing the laparoscopy or administering the PIPAC. Three (75%) patients completed two or more cycles of PIPAC, including 1 (25%) patient that completed six cycles, of which the last 3 cycles were given as compassionate use. Median follow-up was 11.5 months (range 5.4-30.1). One (25%) patient had a prolonged recovery time after the first PIPAC cycle leading to study withdrawal. Two (50%) patients had disease progression following the second PIPAC treatment.

### Safety of PIPAC

There were no Clavien-Dindo surgical complications or DLT. There were ten grade 2 or higher toxicities (one grade 3, nine grade 2) recorded for this cohort of 4 patients, attributable to the treatment (possible/probably/definite). The most common toxicity was abdominal pain (**Supplementary Table 3**). Following PIPAC cycle 1, one patient had grade 2 toxicity and one patient had grade 3 toxicity. The grade 3 abdominal pain toxicity was associated with “Patient 1” who discontinued treatment due to prolonged recovery after her first cycle of PIPAC; her discontinuation of treatment was noted as toxicity. Of note, she had chronic partial SBO symptoms. No grade 4/5 AEs occurred. There were no port-site complications. There was no difference in QOL measures between patients over time. Daily step counts available from 3 patients followed similar patterns with a decrease immediately after surgery and gradually increasing over time until next cycle of PIPAC (**Supplementary Figure 1**).

### Efficacy of PIPAC

**Figure 2:**
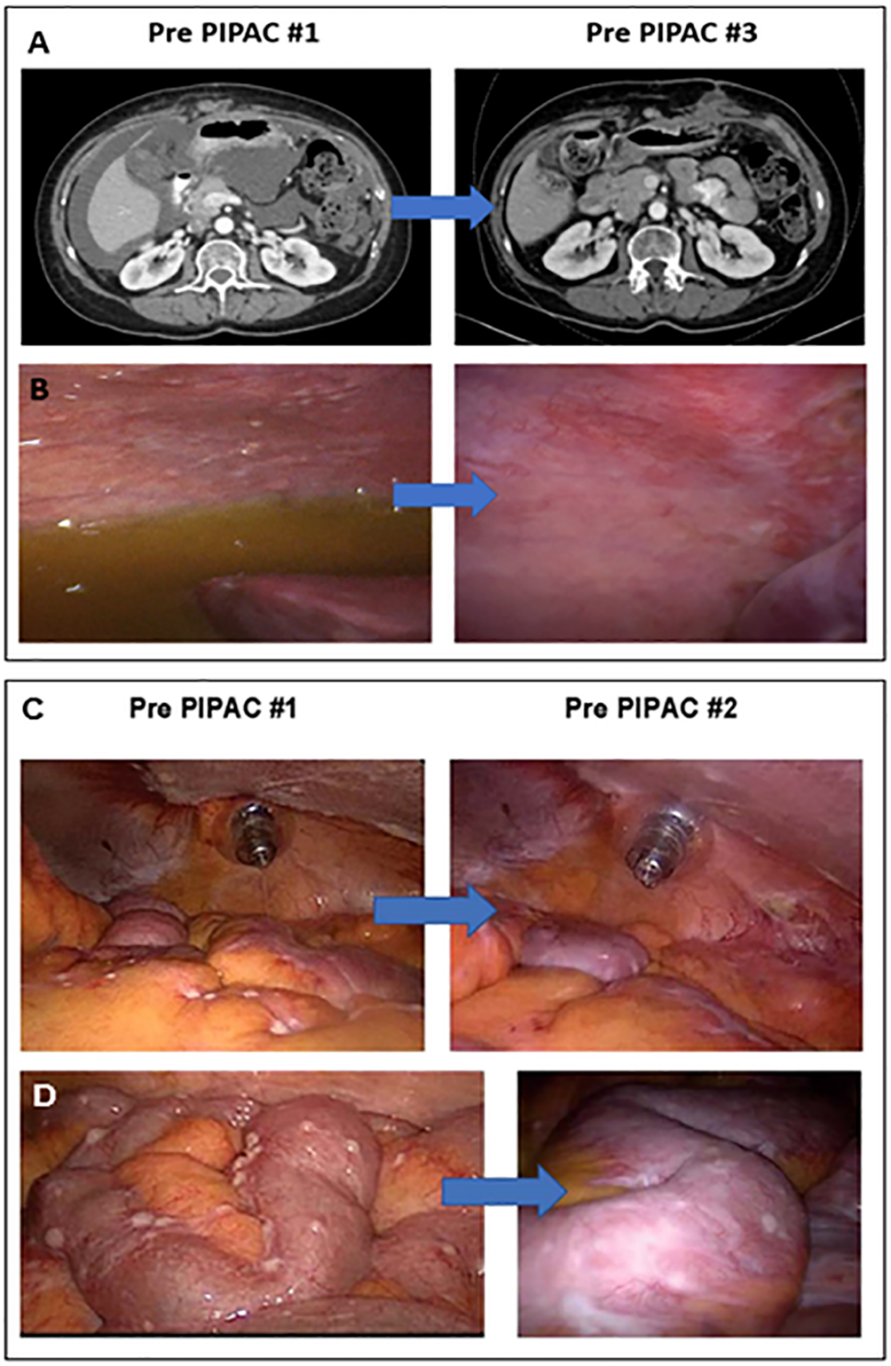
PIPAC treatment in two LGSOC patients. **(A)** “Patient 4” CT scan imaging after two cycles demonstrated a subtotal resolution of ascites with a moderate decrease in peritoneal carcinomatosis; **(B)** “Patient 4” had significant flattening of peritoneal and diaphragmatic nodules, as well as a total resolution of ascites, seen on the laparoscopic assessment performed during PIPAC cycle #3; **(C)** “Patient 3” showed a decrease in the number of nodules evident in the bowel mesentery on the laparoscopic evaluation performed before PIPAC cycle #2 compared to PIPAC cycle #1; **(D)** “Patient 3” showed a post-treatment flattening effect was noted in bowel surface nodules on the laparoscopic evaluation conducted before PIPAC cycle #2 compared to PIPAC cycle #1.

Response to PIPAC treatment was assessed in three ways: CT imaging by RECIST, intraoperative PCI, and pathologic PRGS. Following the first PIPAC cycle, two (50%) patients had a decrease in PCI (**Figure 2**). After two PIPAC cycles by RECIST, “Patient 4” (25%) had a partial response (PR) (**Figures 2A, B**) and “Patient 3” (25%) had progressive disease (PD) based on progression of extraperitoneal and liver parenchymal lesions, but partial response was seen in the peritoneum based on PCI (**Figures 2C, D**).

### Response

**Figure 3:**
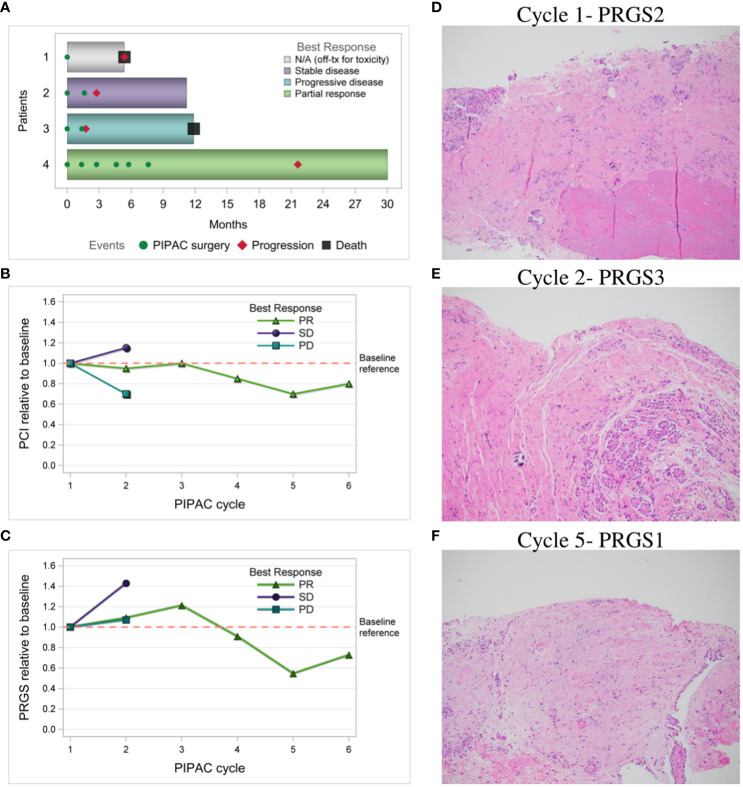
Response to PIPAC treatments. **(A)** Swimmer plot of each patient and best response to treatment measured by CT imaging using RECIST; **(B)** Laparoscopic PCI relative to baseline over PIPAC cycles by best response via RECIST; **(C)** Histologic response relative to baseline by mean PRGS over PIPAC cycles by best response via RECIST; **(D–F)** “Patient 4” PRGS in the left upper quadrant over multiple PIPAC cycles, H&E stained FFPE slides, resolution 10x; **(D)** PIPAC cycle #1 PRGS2 shows infarct-like necrosis (bottom of photo) and dense fibrosis with occasional calcifications (middle), and a small number of nests of viable carcinoma near the surface (top of photo); **(E)** PIPAC cycle #2 PRGS3 about half viable carcinoma (right side of photo) and half treatment-associated dense fibrosis (left side); and **(F)** PIPAC cycle #5 PRGS1 shows only fibrosis with some hemorrhagic areas, and no viable tumor nests or single tumor cells. RECIST, Response Evaluation Criteria in Solid Tumors; PCI, peritoneal carcinomatosis index; PRGS, peritoneal regression grading score; PD, progressive disease; SD, stable disease, PR, partial response; H&E, hematoxylin and eosin; FFPE, formalin**-**fixed paraffin**-**embedded.

ORR was 25% based on measurable intraperitoneal disease at trial entry. **Figure 3A** shows swimmer plot reporting the best response of each patient to treatment measured by CT imaging using RECIST. The change in laparoscopic PCI over each PIPAC cycle by best response via RECIST is shown in **Figure 3B**, with the blue line representing “Patient 3” with PD, green line representing “Patient 4” with PR, and purple line representing “Patient 2” with stable disease (SD). The change in histologic response by mean PRGS over each PIPAC cycle using best response via RECIST is reported in **Figure 3C**. “Patient 4” (25%) shown in **Figures 3D–F** had a decrease in PRGS following three cycles of PIPAC. “Patient 2 and 3” (50%) came off study after two cycles due to PD; “Patient 3” had increase in RECIST and “Patient 2” with best response SD by RECIST but had clinical signs of PD with increasing, symptomatic ascites. In “Patients 2, 3, and 4” who completed at least 2 PIPAC cycles, there was a 5% median decrease in PCI between cycle 1 and 2. Among these three patients, ascites decreased in “Patients 3 and 4” (67%).

### Survival

The median PFS was 4.3 months (range 1.7-21.6). “Patients 2 and 3” who came off study after cycle 2 at 3.2 months and 1.7 months respectively had areas of IP and extraperitoneal disease progression. Of note, both patients had baseline IP and extraperitoneal metastatic disease. “Patient 3” had received 10 prior lines of therapy and had pre-existing thoracic and liver parenchymal metastases. She had significant peritoneal regression (**Figures 2C, D**) as observed by PCI reduction of 20 to 14. However, due to PD of her extraperitoneal and liver parenchymal metastases, she was taken off trial and restarted on IV chemotherapy. “Patient 2” progressed at 3.2 months had received 6 prior lines of therapy and had pre-existing thoracic, breast, and flank metastases. She was noted to have overall SD by RECIST (mixed imaging response in IP region and minimal increase in extraperitoneal metastases), but developed recurrent, worsening ascites, requiring paracentesis treatment, and elected to withdraw from the trial to restart IV chemotherapy. “Patient 1” withdrew from the study for toxicity after cycle 1 had received only 2 prior lines of therapy including letrozole and trametinib, however she was intolerant of this MEK inhibitor. She also had baseline poor ECOG performance status (ECOG=2), chronic partial SBO symptoms, and IP and extraperitoneal metastatic disease. Following withdrawal from the trial, she transferred her care to another provider out of state and no further CT imaging data was available to assess disease status. She died at 5.4 months after starting treatment and this was noted as her date of disease progression for statistical purposes. “Patient 4” had only baseline IP disease without partial SBO symptoms and was noted to have partial response after 3 cycles with reduction in RECIST, resolution of large volume ascites, and normalization of CA 125 (367 to 32), with stable PCI 20. Given her excellent response to therapy, a compassionate use extension was applied, and she received an additional 3 cycles of PIPAC treatment, for a total of 6 cycles of PIPAC. She had further reduction in disease evidenced by reduction in mean PRGS (3.33 to 2.00), PCI (20 to 16), and RECIST over her last 3 cycles. Her DFI was 21.6 months, including 14.0 months following completion of PIPAC treatment.

The median OS was 11.6 months (range 5.4-30.1). “Patients 2 and 4” remain alive and their follow-up time to date is 11.2 and 30.1 months, respectively. Overall survival for “Patient 1” and “Patient 3” were 5.4 and 11.9 months from initiation of PIPAC, respectively.

## Discussion

Treatment options for LGSOC patients are limited, and clinical trials including patients with LGSOC histology are uncommon. Our trial evaluated the role of PIPAC, a novel intraperitoneal chemotherapy method, for regional recurrent disease in LGSOC patients who are not candidates for cytoreductive surgery. This is one of the strengths of this study as few have focused primarily on LGSOC.

Based on safety data from this phase I trial, PIPAC with cisplatin 10.5 mg/m^2^ and doxorubicin 2.1 mg/m^2^ appears to be safe and well tolerated in LGSOC patients without baseline partial SBO symptoms. In our study, no G4/G5 AEs were observed. Overall, our rate of severe AEs (grade 3 or higher) was 25% with one patient having G3 abdominal pain. While we excluded patients with small bowel obstruction, we allowed entry of two patients with partial small bowel obstruction (SBO) who were on limited liquid diet or had chronic nausea and emesis. Unfortunately, these two patients did not tolerate more than 1 or 2 cycles of PIPAC, suggesting a limited role of PIPAC for those patients with partial SBO. Thus, for patients with malignant SBO symptoms, PIPAC may not be well tolerated, likely due to bulkier intraabdominal disease, causing obstruction and poor treatment effect.

Although no other current PIPAC trials have focused on recurrent LGSOC, our observed PFS of 4.3 months and OS of 11.6 months were similar in comparison to the outcomes seen in the PIPAC-OV1 trial, a Phase II trial of platinum resistant recurrent OC patients treated with PIPAC cisplatin/doxorubicin, in which PFS of 4.7 months, and OS of 10.9 months were reported. This trial similarly included a heavily pre-treated population with median lines of therapy 3 (range 2-8) (19). One key difference in patient characteristics was that their trial excluded patients with extraperitoneal disease except for pleural effusion, while our trial included patients with extraperitoneal disease, including lung and liver metastases.

While ORR was measured with RECIST criteria, other measures of peritoneal response were evaluated in our trial, including PCI. Our study demonstrated a decrease in PCI in 66.7% of evaluable patients, which is similar to the decrease seen in the PIPAC-OV1 study, where 76% of patients demonstrated a decreased PCI (19).

In most PIPAC studies to date, histologic regression has been evaluated with a peritoneal regression score called PRGS (18). This grading system was explored in the two Phase II PIPAC OC studies published to date, PIPAC-OV1 and PARROT. However, in PIPAC-OV1, the histologic grading system was based on a neoadjuvant chemotherapy response score rather than PRGS used in in the PARROT and other PIPAC trials (20). The regression rate of 33% in our trial was similar to the PRGS histologic regression of 29.6% in PARROT (21). This contrasts with a histologic regression score of 62% in PIPAC-OV1, where PRGS was not used (19). While PRGS has been used as an endpoint in PIPAC trials, its utility as a primary endpoint has not been universally accepted. Potential bias in PRGS may be introduced by the subjective biopsy selection of surgeons intraoperatively. Additionally, the gross differentiation of normal versus tumor tissue in fibrotic peritoneum can be challenging, contributing to the variability of histologic regression as a reliable measure and universally accepted primary endpoint.

Per the National Comprehensive Cancer Network guidelines and expert consensus report, treatment options for patients with recurrent LGSOC who are not candidates for cytoreductive surgery determined either by imaging or laparoscopic evaluation, include MEK inhibitors, combination MEK and BRAF inhibitors, hormonal therapy, and systemic chemotherapy based on platinum status (3, 22, 23). As the response rate of LGSOC to cytotoxic chemotherapy is <5% in the recurrent setting (24), more effective therapies for these patients are urgently needed. Despite recent advances with MEK inhibitors shown in GOG 281 (ORR 26% trametinib vs 6% standard of care chemotherapy) and MILO/ENGOT-ov11 (16% binimetinib vs 13% physician’s choice chemotherapy), poor tolerance of these drugs limits their role in most LGSOC patients (22, 25). In the NCI-MATCH Trial Subprotocol H looking at BRAF V600E mutated tumors, which included 5 LGSOC patients, the combination of the BRAF inhibitor, dabrafenib with trametinib demonstrated an ORR of 37.9% (26). Anti-estrogen therapy is another alternative to chemotherapy treatment with aromatase inhibitors, tamoxifen, and leuprolide acetate having shown some benefit with ORR 9-14% in the recurrent setting (3). Preliminary data from GOG 3026 combining letrozole with the CDK4/6 inhibitor ribociclib has shown an ORR of 24% (27). However, most of these patients will eventually progress on hormonal therapy. Given the preponderance of peritoneal metastatic disease, regional intraperitoneal therapy may represent a promising novel treatment for LGSOC patients. Our study was limited by a small sample size of four patients, given the rare nature of the disease. Nonetheless, in a heavily pretreated group, a significant intraperitoneal response was demonstrated in two out of three patients who completed PIPAC. Thus, for patients with recurrent disease limited to the IP cavity, and no partial SBO symptoms, further study of PIPAC use in this patient population should be explored. Furthermore, multimodal therapy with systemic chemotherapy in combination with PIPAC could be explored in the future, especially for recurrent LGSOC patients with extraperitoneal and parenchymal tumors. Thus, consideration should be given to future trials which include a combination approach of PIPAC with systemic therapy to improve peritoneal and systemic response in this population of recurrent LGSOC patients.

## Data availability statement

The original contributions presented in the study are included in the article/**Supplementary Material**. Further inquiries can be directed to the corresponding author.

## Ethics statement

The studies involving humans were approved by City of Hope IRB (#19184) Northwell health IRB (#20-0859) Mayo Clinic IRB (#20-010121). The studies were conducted in accordance with the local legislation and institutional requirements. The participants provided their written informed consent to participate in this study.

## Author contributions

BN: Data curation, Formal analysis, Visualization, Writing – original draft, Writing – review & editing. RS: Data curation, Writing – review & editing. NR: Formal analysis, Visualization, Writing – review & editing. PF: Formal analysis, Writing – review & editing. SY: Writing – original draft, Writing – review & editing. SCo: Writing – review & editing. SCh: Methodology, Visualization, Writing – review & editing. AJ: Methodology, Writing – review & editing. ME: Writing – review & editing. RT: Writing – review & editing. DS: Methodology, Writing – review & editing. EW: Methodology, Writing – review & editing. JC: Writing – review & editing. JV: Methodology, Writing – review & editing. RW: Methodology, Writing – review & editing. AM: Methodology, Writing – review & editing. DD: Methodology, Writing – review & editing. MC: Methodology, Writing – review & editing. MW: Methodology, Writing – review & editing. MR: Conceptualization, Methodology, Project administration, Writing – review & editing. TD: Conceptualization, Methodology, Project administration, Writing – review & editing.

## Supplementary material

Supplementary Table 1 Eligibility Criteria.

Supplementary Table 2 Patient Data.

Supplementary Table 3 Adverse Events.
