# Supplementary material for: Pressurized intraperitoneal aerosolized chemotherapy (PIPAC) experience in patients with recurrent low grade serous ovarian carcinoma (LGSOC): sub-cohort report of phase 1 clinical trial
Source: Front Oncol. 2024 Aug 1;14:1404936. doi: 10.3389/fonc.2024.1404936 (PMC11324501; doi:10.3389/fonc.2024.1404936)
Supplement: Supplementary Table 1 — Eligibility Criteria. [file DataSheet_1.docx]

Supplementary Material

**Supplementary Table 1. Eligibility Criteria**

| Inclusion Criteria:   - Documented informed consent of the participant and/or legally authorized representative - Patients must have histologically confirmed ovarian, uterine, gastric, appendiceal or colorectal cancer with peritoneal carcinomatosis - Prior IP chemotherapy is permitted - ECOG performance status ≤ 2 - Absolute neutrophil count (ANC) ≥ 1500/mm^3^ - Platelets ≥ 100,000/mm^3^ - Hemoglobin ≥ 9g/dL - Serum total bilirubin ≤ 1.5x upper limit of normal (ULN) - Alanine aminotransferase (ALT), serum glutamate pyruvate transaminase (SGPT), and aspartate aminotransferase (AST), serum glutamic oxaloacetic transaminase (SGOT) ≤ 2.5x ULN, unless liver metastases are present or unless patient is known to have chronic liver disease (hepatitis) in which case AST and ALT must be ≤ 5x ULN - Alkaline phosphatase ≤ 2x ULN - Serum creatinine ≤ 1.5x ULN, or creatinine clearance ≥ 40 mL/min as calculated by the Cockcroft-Gault formula - No contraindications for a laparoscopy - The peritoneal disease does not have to be measurable by RECIST 1.1 but needs to be visible on cross sectional imaging or diagnostic laparoscopy - Patients must have progressed on at least one evidence-based chemotherapeutic regimen - For patients with a known history of chronic hepatitis B virus (HBV) infection, the HBV viral load must be undetectable on suppressive therapy, if indicated - Patients with a known history of hepatitis C virus (HCV) infection must have been treated and cured. For patients with HCV infection who are currently on treatment, they are eligible if they have an undetectable HCV viral load - Women of childbearing potential (WOCBP) and male patients with WOCBP partner must be using an adequate method of contraception to avoid pregnancy throughout the study and for up to 12 weeks after the last dose of investigational product in such a manner that the risk of pregnancy is minimized. WOCBP include any female who has experienced menarche and who has not undergone successful surgical sterilization (hysterectomy, bilateral tubal ligation, or bilateral oophorectomy) or is not postmenopausal. Post menopause is define as:   - Amenorrhea ≥ 12 consecutive months without another cause or   - For women with irregular menstrual periods and on hormone replacement therapy (HRT), a documented serum follicle stimulating hormone (FSH) level > 35 mIU/mL   - Women who are using oral contraceptives, other hormonal contraceptives (vaginal products, skin patches, or implanted or injectable products), or mechanical products such as an intrauterine device or barrier methods (diaphragm, condoms, spermicides) to prevent pregnancy, or are practicing abstinence or where their partner is sterile (e.g., vasectomy) should be considered to be of childbearing potential - INCLUSION TO PROCEED WITH PIPAC: Laparoscopy findings must meet all of the below criteria in order to proceed to PIPAC:   - PIPAC access is feasible   - There is room for aerosol therapy   - There is no evidence of impending bowel obstruction   - ≤ 5L of ascites   - Not a candidate for cytoreduction and HIPEC |
| --- |
| Exclusion Criteria:   - Previous treatment with maximum cumulative doses of doxorubicin, daunorubicin, epirubicin, idarubicin, and/or other anthracyclines and anthracenediones |

**Supplementary Table 2. Patient Data**

|  | Patient 1 | Patient 2 | Patient 3 | Patient 4 |
| --- | --- | --- | --- | --- |
| Age (years) | 57 | 38 | 68 | 59 |
| Race/Ethnicity | Non-Hispanic White | Non-Hispanic White | Non-Hispanic White | Non-Hispanic White |
| Body Mass Index at Cycle 1 | 15.8 | 25.9 | 32.9 | 24.4 |
| ECOG Performance Status Grade | 2 | 1 | 1 | 1 |
| Prior Lines of Therapy | 2 | 4 | 10 | 6 |
| Baseline Metastatic Status | Extraperitoneal and IP | Extraperitoneal and IP | Extraperitoneal and IP | IP only |
| PIPAC Cycles Received | 1 | 2 | 2 | 6 |
| Baseline PCI | 20 | 33 | 20 | 20 |
| Baseline PRGS | 2.75 | 1.75 | 3.50 | 3.00 |
| Baseline Ascites Volume | 50 mL | 3000 mL | 10 mL | 1500 mL |
| Best Response per RECIST | Unk | SD | PD | PR |
| Percent change in PCI from cycle 1 to 2 | -- | 15% | -30% | -5% |
| PFS Months | 5.4 | 3.2 | 1.7 | 21.6 |
| OS Months | 5.4 | 11.2 | 11.9 | 30.1 |
| Off Treatment Reason | Toxicity | Clinical Progression | RECIST based Progression | Treatment Completed Per Protocol |
| Progression Type | No progression documented | Extraperitoneal and IP | Extraperitoneal and parenchymal | IP |
| *ECOG, Eastern Cooperative Group; IP, intraperitoneal; PIPAC, pressurized intraperitoneal aerosolized chemotherapy; PCI, peritoneal carcinomatosis index; PRGS, peritoneal regression grading score; RECIST, Response Evaluation Criteria in Solid Tumors; Unk, unknown; SD, stable disease; PD, progressive disease; PR, partial response; PFS, progression free survival; OS, overall survival | | | | |

**Supplementary Table 3. Adverse Events**

|  | **Cycle 1** | | **Cycle 2+** | |
| --- | --- | --- | --- | --- |
| **Adverse Event Name** | Grade 2 | Grade 3 | Grade 2 | Grade 3 |
| Abdominal Pain | 1 | 1 | 2 |  |
| Fatigue | 1 |  | 1 |  |
| Hypomagnesemia |  |  | 1 |  |
| Hypotension |  |  | 1 |  |
| Nausea |  |  | 1 |  |
| Vomiting |  |  | 1 |  |
| *Grading by Common Terminology Criteria for Adverse Events (CTCAE) v5.0 | | | | |

**Supplementary Figure 1. Overlay smoothed spline plot of median steps around 3 PIPAC cycles**


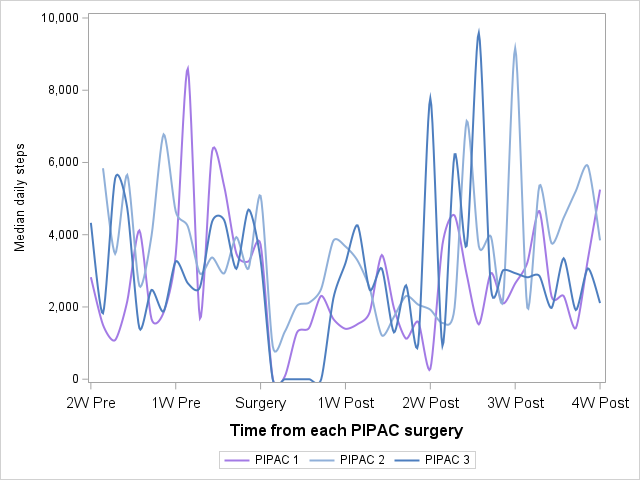


Demonstrates the decline in median steps following each PIPAC surgery, as well as recovery over 4 weeks post-surgery for PIPAC cycles 1-3, as available.
